# Wounds of the Intestines Treated by Suture—Recovery

**Published:** 1844-05

**Authors:** 


WOUND OF THE INTESTINE, TREATED BY SUTURE—RECOVERY.

The following interesting case is contained in a letter from Dr. J. D. McBrayer, of Harrodsburg, Ky., to Prof. Gross.

On the 18th of March, 1843, a negro man (belonging to J. T. of Mercer county), 30 years of age, strong and athletic, received several stabs, one of which penetrated the cavity of the abdomen, midway between the umbilicus and the anterior superior spinous process of the ilium. The wound externally was about three inches in length, but not more than an inch where it penetrated the cavity of the abdomen. I saw him eight hours after the injury was inflicted, and found eighteen inches or two feet of the small bowel protruding. A moist cloth had been laid over the exposed bowel for protection; they had become partially dry and adherent. Warm fomentations were applied and the cloth removed; upon examination of the protruded bowel, a small puncture, about four lines in length, was discovered, penetrating it transversely, the mucous edges being completely inverted. Fortunately, remembering the strict injunctions given in your interesting lectures upon that subject, I adopted the course recommended by you, of closing the puncture by suture where there existed a liability to the discharge of fæcal matter through the wound. A single stitch with a common sewing needle armed with silk, sufficed to close the orifice so as to prevent the escape of the contents of the bowel. With some little difficulty, the bowel was reduced without further dilating the wound. After ascertaining that there was but little if any hemorrhage into the abdomen, the edges of the wound were drawn together, and retained by three stitches, supported by adhesive strips, a compress and roller, &c. The other wounds were dressed by suture, adhesive strips and the bandage.

The patient being considerably exhausted by loss of blood from a wound on the hip involving the gluteal artery, stimulants were administered with freedom, during the dressing and for several hours after. About six hours after the dressing, when he had partially recovered from the immediate effects of loss of blood, the bowels were thoroughly evacuated by an enema of warm water.

March 19th. Circulation rather feeble with general languor and harassing cough, probably induced by remaining for some hours in his bloody clothing,—directed some mild expectorant remedy.

March 20th. Less languor; cough mitigated; pulse has more force and frequency; bowels sufficiently active.

21st. No improvement in the cough; reaction thoroughly established; slight soreness of the bowels; pulse small and tense; directed a saline draught.

22d. Cough continues; complains of pain in the bowels, increased by pressure; pulse hard and 120 to the minute; v. s. to ℥x. when the pulse became soft, full, and less frequent, and the pain in bowels greatly relieved. In the afternoon, the pain returned with the tension and force of the pulse; v. s. to ℥viii.; soon after which all symptoms of peritoneal inflammation subsided. His bowels were regulated by the use of oil and saline purgatives.

On the 25th the dressings were changed; the wounds looked healthy and were healing.

After this the dressings were regularly renewed every two or three days. The stitches closing the wound of the abdomen were not removed until the 15th day, in consequence of a remaining disposition to cough. In twenty days from the time the injury was received, the patient was enabled to walk out, and in six months he resumed his ordinary business. He still remains well.

